# Impairment of Base Excision Repair in Dermal Fibroblasts Isolated From Nevoid Basal Cell Carcinoma Patients

**DOI:** 10.3389/fonc.2020.01551

**Published:** 2020-08-07

**Authors:** Aurélie Charazac, Nour Fayyad, David Beal, Sandrine Bourgoin-Voillard, Michel Seve, Sylvie Sauvaigo, Jérôme Lamartine, Pascal Soularue, Sandra Moratille, Michèle T. Martin, Jean-Luc Ravanat, Thierry Douki, Walid Rachidi

**Affiliations:** ^1^SYMMES/CIBEST UMR 5819 UGA-CNRS-CEA, Univ. Grenoble Alpes, Grenoble, France; ^2^LBFA and BEeSy, PROMETHEE Proteomic Platform, Université Grenoble Alpes, Grenoble, France; ^3^Inserm, U1055, PROMETHEE Proteomic Platform, Saint-Martin-d’Heres, France; ^4^CHU Grenoble Alpes, Institut de Biologie et de Pathologie, PROMETHEE Proteomic Platform, La Tronche, France; ^5^LXRepair, La Tronche, France; ^6^CNRS UMR 5305, Laboratoire de Biologie Tissulaire et Ingénierie Thérapeutique, Lyon, France; ^7^Laboratoire de Génomique et Radiobiologie de la Kératinopoïèse, CEA/DRF/IBFJ/IRCM, Université Paris-Saclay, Evry, France

**Keywords:** nevoid basal cell carcinoma syndrome, *PTCH1* mutation, DNA repair, base excision repair, ROS production

## Abstract

The nevoid basal cell carcinoma syndrome (NBCCS), also called Gorlin syndrome is an autosomal dominant disorder whose incidence is estimated at about 1 per 55,600–256,000 individuals. It is characterized by several developmental abnormalities and an increased predisposition to the development of basal cell carcinomas (BCCs). Cutaneous fibroblasts from Gorlin patients have been shown to exhibit an increased sensitivity to ionizing radiations. Mutations in the tumor suppressor gene *PTCH1*, which is part of the Sonic Hedgehog (SHH) signaling pathway, are responsible for these clinical manifestations. As several genetic mutations in the DNA repair genes are responsible of photo or radiosensitivity and high predisposition to cancers, we hypothesized that these effects in Gorlin syndrome might be due to a defect in the DNA damage response (DDR) and/or the DNA repair capacities. Therefore, the objective of this work was to investigate the sensitivity of skin fibroblasts from NBCCS patients to different DNA damaging agents and to determine the ability of these agents to modulate the DNA repair capacities. Gorlin fibroblasts showed high radiosensitivity and also less resistance to oxidative stress-inducing agents when compared to control fibroblasts obtained from healthy individuals. Gorlin fibroblasts harboring *PTCH1* mutations were more sensitive to the exposure to ionizing radiation and to UVA. However, no difference in cell viability was shown after exposure to UVB or bleomycin. As BER is responsible for the repair of oxidative DNA damage, we decided to assess the BER pathway efficacy in Gorlin fibroblasts. Interestingly, a concomitant decrease of both BER gene expression and BER protein activity was observed in Gorlin fibroblasts when compared to control. Our results suggest that low levels of DNA repair within Gorlin cells may lead to an accumulation of oxidative DNA damage that could participate and partly explain the radiosensitivity and the BCC-prone phenotype in Gorlin syndrome.

## Introduction

First described by Gorlin and Goltz in 1960 (1), the nevoid basal cell carcinoma syndrome (NBCCS), also called Gorlin syndrome, is a rare autosomal dominant disorder whose prevalence varies from 1 case per 55,600–256,000 in the general population (2). Patients with NBCCS may present various developmental abnormalities such as palmar and plantar pits, odontogenic keratocysts, skeletal anomalies (bifid ribs, scoliosis, polydactyly), neurological disorders (like mental deficiency), and an increased predisposition to cancers including medulloblastoma and basal cell carcinomas (BCCs) (2, 3). The onset of skin tumors is at a young age (3) in contrast to that observed at 60–65 years in the general population. NBCCS patients are predisposed to radio-induced cancers. Indeed, Gorlin patients undergoing radiotherapy treatment may develop multiple BCCs, within the irradiation field (scalp and neck) 6 months to 3 years after treatment (4). In addition to the occurrence of BCCs, Gorlin patients are at high risk of developing medulloblastoma which is the cause of premature death, rhabdomyosarcoma, ovarian, and uterine fibromas (3).

To date, 539 heterozygous mutations have been reported in the tumor suppressor gene *PTCH1* which is mainly considered to be responsible for Gorlin syndrome (3, 5). Protein patched homolog 1 (PTC1), the protein encoded by this gene, acts as a transmembrane receptor for the sonic hedgehog (SHH) ligand (6). In the absence of SHH, PTC1 constitutively suppresses the activation of the hedgehog signaling by inhibiting a second transmembrane protein called Smoothened (SMO). The binding of SHH to PTC1, or PTC1-inactivating mutations, relieves this suppression and restores SMO activity, which leads to the activation and nuclear translocation of the zinc transcription factors (GLI1, GLI2, and GLI3) (7). Highly conserved among species, this SHH signaling pathway plays a critical role in patterning, proliferation, and cell fate determination of a broad range of cells and tissues (8). In the adult stage, the pathway is considerably down-regulated and is restricted to tissue regeneration and stem cell renewal (9). SHH aberrant activation has been observed in several types of human cancer such as leukemia, gastrointestinal, lung, ovarian or even breast and in almost all sporadic BCCs (10).

NBCCS patient cells exhibit chromosomal instability (11). Sensitivity to genotoxic stresses such as UV (12, 13) and ionizing radiation (14) have been reported. However, contradictory results have been published on this hypersensitivity for UVA, UVB, and IR (15, 16). Thus, the underlying mechanism remains elusive in the Gorlin patients. One explanation might be given by Vulin et al. (17) who demonstrated that the variable radiosensitivity of Gorlin patients was correlated to the type of *PTCH1* mutation and the level of PTC1 defect. The authors also pointed out defective DNA damage response signaling (DDR), notably concerning the phosphorylation of CHK2 and P53. Other studies have suggested that increased radiosensitivity of cells might be strongly linked to their inability to repair the DNA lesions induced by the exposure to radiation (18–20). Because complex DNA damages and in particular double-strand breaks play a major role in the lethality and mutagenicity of ionizing radiation, deficient DDR and DNA repair may be one of the origins of NBCC.

A wide variety of DNA repair pathways are active in cells. They evolved in order to maintain the integrity of the genome that is frequently threatened by both endogenous and exogenous stresses. Among all, base excision repair (BER) pathway is responsible for repairing small DNA lesions such as deamination, alkylation or single-strand breaks. Above all, it is accountable for repairing reactive oxygen species (ROS) or oxidative stress induced-lesions, as 8-oxoguanine (8-oxoG). 8-oxoguanine is the most extensively studied DNA lesion where up to 100,000 lesions are estimated to be formed in DNA per cell daily and could be implicated in BCC carcinogenesis if left unrepaired (21–23). BER pathway is initiated by one of at least 11 distinct mammalian DNA N-glycosylases (like OGG1 and MYH) that recognizes and removes specific damaged base, generating an abasic site (AP), which is subsequently cleaved by an AP endonuclease. The resulting single-stranded break can then be processed by the combined action of polymerase and ligase (24, 25). Nucleotide excision repair (NER) is an additional important mechanism that handles a variety of lesions affecting the DNA helix structure, including UV-induced lesions and bulky chemical adducts. The NER process involves the action of more than 30 proteins in a stepwise manner that includes damage recognition, local unwinding of the helix, dual incision of the lesion, gap-filling using the undamaged single-stranded DNA and finally, the strand ligation (24, 26). Other important pathways are the homologous recombination (HR) and the non-homologous end joining pathway (NHEJ) both allowing the repair of DNA double-stranded breaks notably induced by ionizing radiation (24, 27).

Like NBCCS, numerous other genetic disorders present hyper-radiosensitivity, hyper-photosensitivity or even increased proneness to cancer. This is the case for Xeroderma Pigmentosum (XP) where a mutation in one of the DNA repair genes of the NER pathway leads to UV sensitivity, neurological abnormalities and a great risk of developing skin cancer (28). Another such genetic disorder is Ataxia-Telangiectasia (AT) which is characterized by hypersensitivity to ionizing radiations, predisposition to malignancies and neurological defects because of mutations in the ATM gene involved DDR, including NHEJ repair pathway (29). Since a deficiency in DNA repair is a common feature to these syndromes, we decided to investigate whether this was also the case in Gorlin syndrome. Therefore, we designed this work in order to study the sensitivity of skin fibroblasts from NBCCS patients to different DNA damaging agents and the modulation of the DNA repair capacities. We demonstrated that the *PTCH1* mutations present in Gorlin fibroblasts lead them to be more sensitive to the exposure to ionizing radiation and to UVA. Furthermore, our experiments showed a drastic decrease in the gene expression of BER and DNA damage signaling. Besides, the BER activity was found to be globally downregulated in these *PTCH1* mutated cell lines. Finally, an increased amount of superoxide anion was found in Gorlin fibroblasts after an irradiation stress.

## Materials and Methods

### Cell Lines and Culture Conditions

Non-immortalized human dermal fibroblasts of NBCCS patients from 11 to 31 years old (GM01552, GM02098, and GM03300) were purchased from the Coriell Institute for Medical Research (NJ, United States). Mutations of GM01552 and GM02098 have been described in (17). Mutations of GM03300 have been now characterized and are presented in Supplementary Table S2.

Fibroblasts from a healthy 45 years old (GM00730) was found to be representative amongst two other control human primary fibroblasts (GM0116 and GM0121) as shown in the Supplementary Figure S1. The Supplementary Table S1 summarizes all the information on cell strains.

The cells were grown in Dulbecco’s modified Eagle’s medium supplemented with Glutamax (Invitrogen, CA, United States), 10% FBS (Thermo Fisher Scientific, IL, United States) and 1% penicillin-streptomycin (Invitrogen, CA, United States). Incubation was routinely done at 37°C in an incubator supplied with 5% CO_2_.

The three Gorlin fibroblast GM03300, 1552, and 2098 strains were obtained from the Coriell Institute. The proliferative capacity of each cell strain has been characterized in terms of doubling time over several passages in culture, to check the stability of cell proliferation and avoid using cells entering senescence. In our culture conditions, the mean doubling time of the control cells was 30.17 ± 1.03. For the Gorlin cells, doubling times were 46.76 ± 4.95, 47.35 ± 1.95 and 33.66 ± 1.88, respectively. For all fibroblast cell strains, five mean population doublings were obtained at each passage.

### Short-Term Cytotoxicity Assay

Short-term cytotoxicity of gamma irradiation (2.2 Gy/min, Co^60^, Arc Nucleart, CEA Grenoble) was tested with ranging doses from 0 to 10 Gy.

Fibroblasts were plated at a density of 10,000 cells/cm^2^ for the MTT (3-[4,5-dimethylthiazol-2-yl]-2,5 diphenyl tetrazolium bromide) assay. This assay is based on the conversion of MTT into formazan crystals by living cells. The next day, the medium was changed and the cells were exposed to gamma irradiation. 24 h after, 0.5 mg/mL of MTT is added to each well and incubated at 37°C for 4 h. Then, after discarding the medium in the wells, DMSO was added and the absorbance was recorded at 570 nm using a microplate reader.

### Long-Term Cytotoxicity Assay

Long-term cytotoxicity of gamma irradiation (2.2 Gy/min, Co^60^, Arc Nucleart, CEA Grenoble), UVA radiation (UVA 700L Waldmann, Germany) and UVB radiation (VL215G irradiator, Bioblock, France) were tested with ranging doses from 0 to 10 Gy, 0 to 100 J/cm^2^, and 0 to 0.025 J/cm^2^, respectively. Bleomycin was also tested as pure DNA double-stranded breaks producer with doses varying from 0 to 1 μM.

Fibroblasts were plated at a density of 20 cells/cm^2^ for the clonogenic assay. The next day, the medium was changed and the cells were exposed to different stress agents. Cells were cultured as previously described (30). They were then fixed and stained with crystal violet, in order to count the number of colonies by microscopy.

### Treatment and Frozen Pellets Preparation

Subconfluent cells were either not exposed (0 Gy) or exposed to 6 Gy gamma radiation at a dose rate of 2.2 Gy/min. Fibroblasts were harvested either 4 h (RT-qPCR assay) or 24 h (DNA repair activity assay) after irradiation, centrifuged and rinsed with PBS (Invitrogen, CA, United States). Then, pellets were rapidly frozen at −80°C until further use.

### Reverse Transcription and Real-Time Quantitative PCR (RT-qPCR) Analysis

Total cellular RNA was extracted from each sample using the GenElute^TM^ Mammalian Total RNA Miniprep kit (Sigma-Aldrich, Saint-Quentin Fallavier, France) according to the manufacturer’s protocol with the optional DNase treatment step. RNA quantification was done using NanoDrop^TM^ 1000 Spectrophotometer (Thermo Fisher Scientific, IL, United States) and its quality was assessed using native agarose gel electrophoresis. Total RNA was considered intact when two acute 28S and 18S bands were visualized.

For each condition, 2 μg of RNA were reverse transcribed to cDNA (Superscript^®^ III Reverse Transcriptase, Invitrogen, CA, United States) in the presence of 100 ng/μL of random primers (Promega, Charbonnières, France), dNTP mix (10 mM of each, Sigma-Aldrich, Saint-Quentin Fallavier, France), 5X-First strand buffer, 0.1 M DTT, ribonuclease inhibitor (Sigma-Aldrich, Saint-Quentin Fallavier, France) and SuperScript III enzyme (Invitrogen, CA, United States).

Ten ng of each cDNA was used in qPCR reactions with gene-specific primers (Supplementary Table S3). qPCR was performed in a MX3005p Multiplex Quantitative PCR System (Stratagene, CA, United States) using Takyon^TM^ qPCR MasterMix for SYBR^®^ assays containing Low ROX passive reference (Eurogentec, Angers, France). At the end of each run, the amplification’s integrity indicated by a single melt peak for each product was verified using a dissociation curve analysis. Target gene mRNA expression was normalized to the expressed housekeeping gene glyceraldehyde-3-phosphate dehydrogenase (GAPDH). The quantification was done using the comparative Ct method involving the comparison of the Ct values of the sample of interest with the Ct values of normal non-irradiated RNA as calibrator. The target’s amplifications efficiencies as well as the endogenous references were considered to be approximately equal.

### DNA Excision-Synthesis Repair Activity Analysis

The DNA repair biochips measured the excision-synthesis repair activities of substrates present in supercoiled plasmid DNA, immobilized on support. Repair activity is reflected by the incorporation of fluorescent nucleotides (dCTP-Cy5) at the lesions sites. Five lesions-containing plasmids known to be repaired by distinct enzymes and/or pathways (BER and NER) were located at predetermined sites on the biochips: photoproducts, 8-oxoguanine, alkylated bases, pyrimidine glycols and APs. The substrates were controlled by HPLC-MS/MS (31). Images were acquired by scanning the biochip at 532 nm using an InnoScan710AL scanner (Innopsys, France). Total spot fluorescence intensity was quantified using the Mapix software (Innopsys, France). Data were normalized using NormalizeIt software (31). Each sample was characterized by five values corresponding to the repair of the five DNA lesions present on the biochip.

As described in Millau et al. (31), nuclear extracts were prepared and used at 0.4 mg/mL to conduct the DNA repair excision-synthesis reaction on the modified plasmid arrays. Each extract was tested in duplicate.

### Mitochondrial Superoxide Production Analysis

Mitochondrial superoxide generation was assessed using MitoSOX^TM^ (Life Technologies, CA, United States). This fluorogenic dye is selectively targeted to the mitochondria of live cells where it is oxidized by superoxide but not by other ROS. MitoSOX^TM^ was added at a final concentration of 5μM according to the manufacturer’s recommendation. Cells were allowed to load this dye for 10 min and then washed with PBS (Invitrogen, CA, United States). The measurements were carried out using a FACScalibur instrument (BD Biosciences, United States).

### Statistical Analysis

The data were expressed as the mean ± SD of three different experiments. Statistical significance of data was assessed using the student’s *t*-test (Microsoft Excel) after checking variance homogeneity with the Levene’s test or one-way ANOVA as appropriate. Differences were considered significant when the *p*-value was < 0.05.

## Results

### NBCCS Fibroblasts Are Specifically More Sensitive to Ionizing Radiation and UVA-Induced Cytotoxicity

**FIGURE 1:**
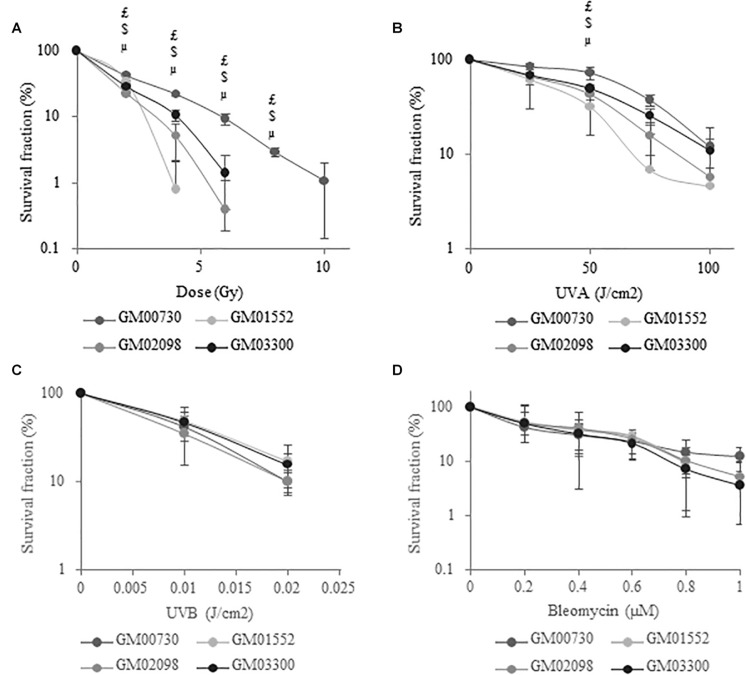
NBCCS fibroblasts are specifically more sensitive to ionizing radiation and UVA-induced cytotoxicity. Long-term survival curves determined by clonogenic assays. Cells were plated at low density (20 cells/cm^2^) and the second day they were exposed to growing dose of **(A)** ionizing radiation (γ Rays), **(B)** UVA radiation, **(C)** UVB radiation or **(D)** bleomycin and cultured for 2 weeks. Cells were then fixed with ethanol, stained with crystal violet in order to count the number of colonies. Results are expressed as mean percentage of survival fraction ± SD of colonies of 3 independent experiments (*n* = 3) and triplicate measurements. μ GM01552 significantly (*p* < 0.05) different from the normal cells (GM00730) stressed at the same dose. $ GM02098 significantly (*p* < 0.05) different from the normal cells (GM00730) stressed at the same dose. $ GM03300 significantly (*p* < 0.05) different from the normal cells (GM00730) stressed at the same dose.

Primary cell cultures were exposed to increasing doses of gamma rays, UVA and UVB radiations, or to increasing concentrations of bleomycin. Long-term survival was then determined 14 days post-exposure using a clonogenic survival assay (30). Using the one-way ANOVA test, we showed that NBCCS fibroblasts are less resistant to ionizing (Figure 1A) and UVA (Figure 1B) radiations when compared to control. However, there was no significant difference in cell toxicity after exposure to UVB (Figure 1C) or bleomycin (Figure 1D). The LD_50_ was thus determined for the four fibroblast lines (Supplementary Table S4) using a clonogenic test. A significant difference was found after ionizing radiation or UVA stress between the normal fibroblast cell line and the three Gorlin fibroblast cell lines. Looking at the results after a UVB radiation or bleomycin exposition did not result in different survival responses between Gorlin and normal fibroblasts.

### Fibroblasts From NBCCS Patients Show an Overall Downregulation of BER and DNA Damage Signaling Genes

**FIGURE 2:**
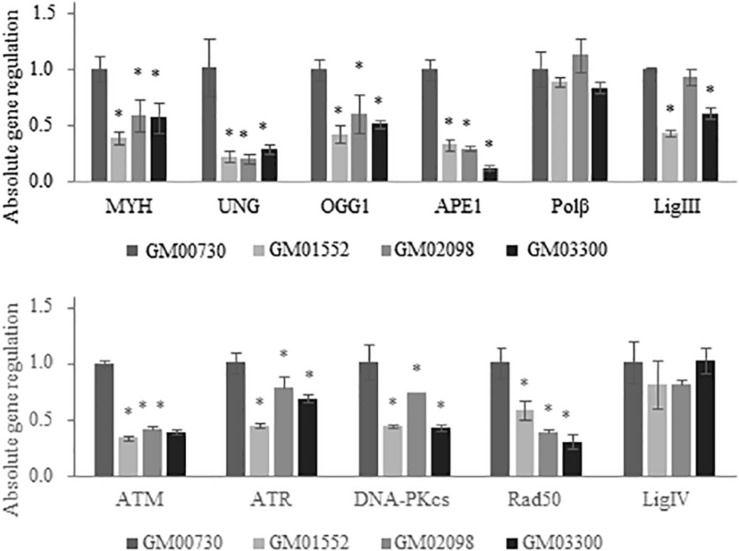
Expression of BER and DNA damage signaling-associated genes in NBCCS fibroblasts at basal level. Gene expression was investigated in Gorlin vs. control fibroblasts at basal level by RT-qPCR experiments. After total RNA extraction of each cell culture, they were retro-transcribed in complementary DNA. 20 ng of cDNA were used to detect specific gene expression. Three independent experiences were realized (*n* = 3). The calibrator used is non-irradiated GM00730. The upper panel reports results for the BER genes while the lower one shows data for the DNA double strand breaks repair. Collapse of BER glycosylases gene expression as well as DNA Damage Response gene signaling is shown here. No statistical differences in the expression of the polymerase beta was found. * Significantly (*p* < 0.05) different from the normal cells (GM00730).

We first examined the expression levels of a large series of DNA repair enzymes in the normal and the three Gorlin primary cell cultures under basal conditions without any exogenous stress using real-time PCR (Figure 2). Several genes from BER, NHEJ, and DNA damage signaling pathways were studied. Numerous BER-associated genes were significantly less expressed in Gorlin cells compared to control. This is the case for *MYH* which is a BER glycosylase that removes misincorporated adenines in front of 8-oxoguanine (8-oxoG) during DNA replication. The expression of the uracil-DNA glycosylase (*UNG*) is also drastically decreased in Gorlin as compared to the normal cells. Similarly, mRNA level of both the human 8-oxoG-specific glycosylase, *OGG1*, and the apurinic endonuclease 1 (*APE1*) expression are reduced in Gorlin cells as compared to control fibroblast. The mRNA expression of polymerase beta (*POL*β*)* is not significantly different in Gorlin and normal fibroblasts. However, ligase 3 (*LIG3*) expression was reduced in GM01552 and GM03300, but no significant difference was found for the GM02098 cell culture.

Next, we assessed the mRNA expression of several DNA damage signaling genes like *ATM* (ataxia telangiectasia mutated gene) which is a kinase recruited and activated by DNA double-strand breaks, *ATR* (ATM and Rad3-related), DNA-dependent protein kinase (*DNA-PKcs*) responsible for the alignment of broken ends of DNA in the NHEJ pathway and *RAD50* who play a role in the early DNA double-strand breaks repair. They were all found decreased in Gorlin fibroblasts as compared to the control. Finally, no significant difference was found between Gorlin and normal cells for the ligase 4 (*LIG4*) expression.

### Effect of Ionizing Radiation on BER-Associated Gene Expression in Normal and Gorlin Fibroblasts

**FIGURE 3:**
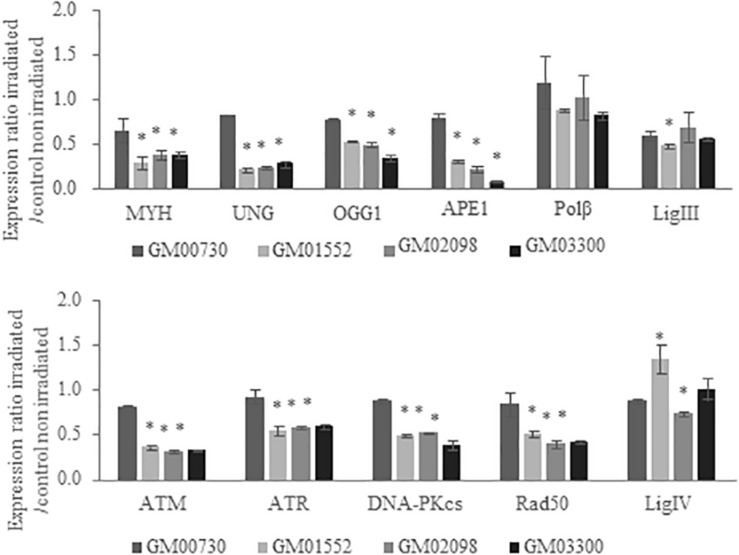
Expression of BER and DNA damage signaling-associated genes in NBCCS fibroblasts after irradiation. Gene expression was investigated by RT-qPCR experiments in NBCCS *vs.* control fibroblasts at 4 h after 6 Gy ionizing irradiation. After total RNA extraction of each cell line, they were retro-transcribed in complementary DNA. 20 ng of cDNA were used to detect specific gene expression. Three independent experiences were realized (*n* = 3). The calibrator use is non-irradiated GM00730. The upper panel reports results for the BER genes while the lower one shows data for the DNA double strand breaks repair. Collapse of BER glycosylases gene expression as well as DNA Damage Response gene signaling are demonstrated here. No statistical differences in the expression of the polymerase beta was found. Furthermore, gene expression of the normal cell line was decreased after a stress. *Significantly (*p* < 0.05) different from the irradiated normal cells (GM00730).

MTT assays (which reflect the short-term toxicity) demonstrate that this stress leads to less than 20% of mortality 24 h after irradiation for both normal and Gorlin fibroblasts (Supplementary Figure S2). We then investigated the gene expression profile of these cell lines 4 h following a 6 Gy irradiation stress (Figure 3). The fold change expression of mRNA level of *MYH*, *UNG*, *OGG1*, and *APE1* was found decreased in the three Gorlin cell cultures as compared to the control. However, no significant differences were found in the gene expression of *POL*β and *LIG3* between Gorlin and normal cells; except for the GM01552 *LIG3* expression.

Regarding *ATM*, *ATR*, *DNA-PKcs*, and *RAD50* expressions, they were downregulated in Gorlin as compared to normal fibroblast. Finally, *LIG4* expression was increased in GM01552 and decreased in GM02098, but no significant difference was found for the GM03300 cells.

As we used the non-irradiated WT cells as a calibrator, the reduction of the expression of BER-associated genes in Figure 3 reproduces the effects observed without irradiation in the Gorlin cells. These results showed the non-adaptability of Gorlin cells to irradiation stress despite their low level of expression of genes associated with the BER.

### Low Intrinsic Base Excision Repair Capacities in NBCCS Fibroblasts

To obtain further information about the BER activity in different fibroblast cultures, we used a specific multiplexed functional assay that allows the analysis of the excision-synthesis repair activities from different damaged DNA substrates immobilized on a biochip (31). Repair activity was reflected by the incorporation of fluorescent nucleotides at the lesion sites. Different supercoiled plasmids containing lesions known to be repaired by BER pathway were used: 8-oxoguanine (8-oxoG), alkylated bases, and cytosine and thymine glycols. The 8-oxoG lesion highlights the repair capacity of the OGG1 (8-oxoguanine glycosylase) glycosylase followed by endonuclease, polymerase and ligase steps. The alkylated lesions show the repair capacity of the alkyladenine DNA glycosylase (AAG) excision activity. Finally, the thymine glycols lesions allow the assessment of the repair capacity of the Thymine DNA glycosylase (TDG).

**FIGURE 4:**
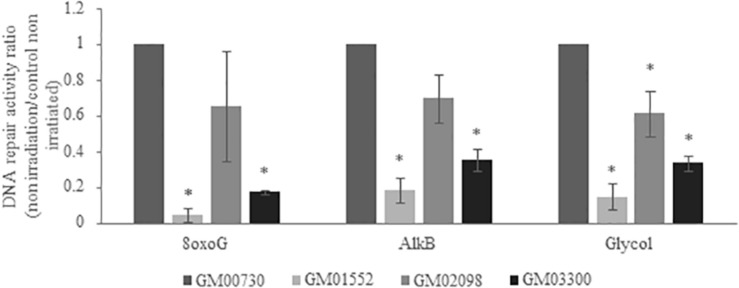
Basal DNA excision-synthesis activity in NBCCS fibroblasts. It was determined using the multiplexed plasmid biochips comparing the normal primary human fibroblasts and the three NBCCS fibroblasts at basal level. The ratio was done with respect to the normal non-irradiated cell line. The 8-oxoG lesions highlight the repair capacity of the OGG1 glycosylase followed by endonuclease, polymerase and ligase steps. The alkylated lesions demonstrate the repair capacity of the alkyladenine DNA glycosylase (AAG) excision activity. Finally, the thymine glycols lesions allow the assessment of the repair capacity of the Thymine DNA glycosylase (TDG). 8-oxoG, AlkB and Glycol were found to be less repaired in Gorlin fibroblasts. *Significantly (*p* < 0.05) different from the normal cells (GM00730).

We first investigated the excision-synthesis activity using the global repair efficiency index of the four cell cultures at the basal level without irradiation (Figure 4). The excision-synthesis capacity of the 8-oxoG lesion was drastically downregulated in GM01552 and in GM03300 when compared to the normal fibroblasts. Once again, the repair capacity of the alkylated bases was reduced in GM01552 and the GM03300. However, no significant difference in the repair activity of 8-oxoG lesion or alkylated bases was found in GM02098 when compared to the normal GM00730 cells. Finally, regarding the excision-synthesis capacity of the thymine glycols, all three Gorlin cell cultures appeared to have a downregulation of this activity when compared to the normal cells.

**FIGURE 5:**
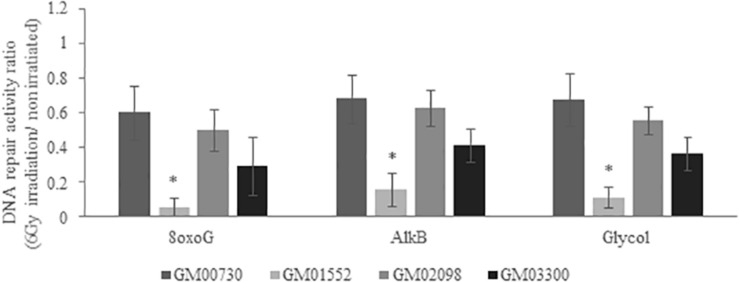
DNA excision-synthesis activity in NBCCS fibroblasts after irradiation. It was determined using the multiplexed plasmid biochips comparing the normal human fibroblasts and the three NBCCS fibroblasts at 24 h after 6 Gy irradiation. The ratio was done with respect to the non-irradiated cell line, respectively. The 8-oxoG lesions highlight the repair capacity of the OGG1 glycosylase followed by endonuclease, polymerase and ligase steps. The alkylated lesions show the repair capacity of the alkyladenine DNA glycosylase (AAG) excision activity. Finally, the thymine glycol lesions allow the assessment of the repair capacity of the Thymine DNA glycosylase (TDG). Once again, 8-oxoG, AlkB and Glycol were found to be less repaired in Gorlin fibroblasts. It is noteworthy that the excision-resynthesis activity of GM00730 after a stress was decreased as compared to the same cell line without irradiation. *Significantly (*p* < 0.05) different from the irradiated normal cells (GM00730).

We then decided to test the excision-synthesis activity of these cells 24 h after a 6 Gy irradiation (Figure 5). The one-way ANOVA statistical analysis demonstrates that the excision-synthesis activity was weakened in the irradiated normal fibroblasts as compared to the non-irradiated (*p*-value = 0.007). In the Gorlin fibroblasts, one-way ANOVA analysis did not show any differences in the excision-synthesis activity of the three studied lesions after a 6 Gy irradiation as compared to the cells without irradiation.

We next compared the repair activities between the irradiated GM00730 cells and the three irradiated Gorlin cells. Globally, all excision-synthesis activities tested were found to be drastically reduced in GM01552 as compared to the control after irradiation while no statistical differences were found for the two other Gorlin fibroblast cultures.

### Superoxide Production Is Increased in Gorlin Fibroblasts

**FIGURE 6:**
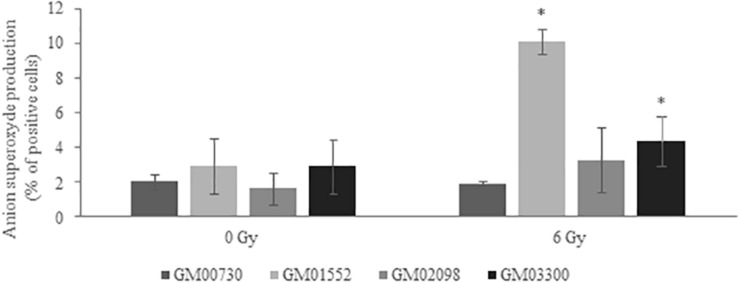
Anion superoxide production in NBCCS fibroblasts at basal level and after irradiation. It was determined 25 min post-stress using MitoSOX^TM^ at 5 μM final concentration. Cells were analyzed by FACS with 510/580 nm excitation/emission conditions. Three experiences were realized (*n* = 3). No significant differences in the production between all cell cultures at basal level was found. On the contrary, GM01552 and GM03300 produced more superoxide anion than the GM00730 after a 6 Gy ionizing radiation. *Significantly (*p* < 0.05) different from the normal cells (GM00730).

MitoSOX^TM^ dye was used to reveal the production of superoxide by mitochondria of the four human primary fibroblasts. A one-way ANOVA analysis revealed that this production was not statistically different between all fibroblast cultures (*p* = 0.37) at basal level. However, GM01552 and GM03300 produced more superoxide anion than the normal cells 25 min after exposure to 6 Gy of γ radiation (Figure 6). The GM02098 cells appear to possess the same ability to produce superoxide as the GM00730 cells.

## Discussion

It has been shown that several cancer-prone diseases result from a defective DNA repair capacity. This is well known for the XP syndrome where patients exhibit a high sensitivity to UV radiation. The abnormal high rate of BCCs in sun-exposed skin is due to mutations in proteins belonging to the NER pathway (28). AT is another example of such kind of disorders. The mutation in *ATM* gene is responsible for the highly increased incidence of lymphomas and leukemia in these patients (29). Thus, the aim of our study was to assess the modulation of several DNA repair systems in NBCCS, a hyper-radiosensitive disorder and cancer-prone disease in order to decipher if Gorlin phenotype is linked to a defect in DNA repair capacity.

Interestingly, this syndrome is related to heterozygous mutations, whereas XP and AT are related to homozygous mutations. The Gorlin cellular model thus opens a field of research which is less investigated, and is still opened to debate (HPA report, 2013).

Our results show that the *PTCH1* mutations lead Gorlin fibroblasts to be more radiosensitive to ionizing radiation and UVA-induced cytotoxicity. Further experiments have shown a drastic decrease in the gene expression of BER and DNA damage signaling genes, as well as in the excision/resynthesis activity of the BER pathway. Finally, an increased level of superoxide anion (a major product of the primary oxidase sources of ROS) was found in Gorlin fibroblasts after exposure to ionizing radiation.

Because Gorlin patients are known to be clinically hyper-radiosensitive to ionizing radiation, we first decided to test the intrinsic radio-sensitivity of the Gorlin cells studied, in order to know if they were able to reflect the NBCCS phenotype. The clonogenic assay showed that the three Gorlin fibroblasts were more sensitive to ionizing radiations. This result is consistent with that obtained by Chan et al. (14). On the contrary, Featherstone et al. (16) were not able to observe a survival difference between NBCCS and control fibroblasts exposed to ionizing radiation. These dissimilarities might be explained by the degree of PTC1 deficiency which is variable among patient cells. Indeed, Vulin et al. (17) showed that only severely decreased gene expression correlates with significantly increased intrinsic radiosensitivity.

Other DNA damaging agents, leading to the formation of other classes of DNA lesions, were also tested. UVA is known to induce DNA damage by its ability to generate CPD (32) and also ROS, which leads to the formation of SSB and oxidized bases such as 8-oxoG (33). We observed that the Gorlin fibroblasts were more sensitive to UVA when compared to the normal ones. We then tested UVB, a CPD and 6–4 PP inducer that are handled by the NER repair pathway. As Brellier et al. (15) have shown, the Gorlin cells were not more sensitive to this agent than control cells. On the contrary, Applegate et al. (12) described hypersensitivity of NBCCS fibroblasts to UVB but they mentioned that the sunlamp used in their experiments substantially emitted in the UVA wavelengths. Finally, we did not observe an increased sensitivity of Gorlin cell lines to bleomycin, a radiomimetic agent producing double-strand breaks through an oxidative stress-independent pathway. In summary, clonogenicity assay demonstrated a strong radiosensitivity and a lower resistance of the Gorlin fibroblasts to oxidative stress agents when compared with the normal fibroblasts. However, no difference was observed against bleomycin and UVB-induced cytotoxicity, indicating that Gorlin fibroblasts are very sensitive to ROS-induced cytotoxicity.

In order to know if the sensitivity to IR and UVA was due to a failure to repair DNA damage induced by these genotoxic agents, we decided to assess the expression of DNA repair genes in all four cell lines at both basal level and after exposure to ionizing radiation. A global downregulation of almost all DNA repair genes was found with a collapse for the excision-synthesis step of BER. Interestingly, *OGG1* and *MYH*, both of which are involved in the 8-oxoG repair, were significantly downregulated at basal level and after exposure to IR. Some studies have already demonstrated that a low expression of *OGG1* might result in the accumulation of ROS generating 8-oxoG lesions (21). Our results also establish a collapse of *UNG* and *APE1* expression. These two glycosylases have been shown to be linked to radiosensitivity (34, 35). Interestingly, it has been proposed by Sobol et al. (36) that a deletion in polymerase beta leads to BER repair deficiency and an hyper-sensitivity to DNA-alkylating agents. Our RT-qPCR experiments did not show any impairment of expression of this polymerase.

ATM and ATR are key mediators of the DDR enabling through their activation the initiation of signaling pathways regulating apoptosis, cell cycle progression and DNA repair (37). The mRNA expression of both kinases was found reduced at basal level and after exposure to ionizing radiation. Studies on mice have shown that the disruption of ATR might lead to chromosomal fragmentation and early embryonic lethality (38, 39). ATM and ATR are key controllers of the DDR on which depends the genome’s stability (40). Moreover, Barzilai et al. (41) have linked the ATM deficiency to ROS regulation. Thus, the downregulation of *ATM* and *ATR* gene expression found here might introduce a genomic instability in Gorlin fibroblast.

Next, we wanted to know if this downregulation was also found at the activity level. In order to answer this question, we used a miniaturized assay allowing the fast and reliable questioning of the excision-synthesis step of the BER repair pathway. Overall, the activity of BER in NBCCS cell lines was decreased when compared to the normal cells line. The repair of alkylated bases and thymine glycols has been found downregulated in NBCCS fibroblasts when compared to controls. Moreover, two of the three NBCCS cell lines presented a decrease in the 8-oxoG lesions excision/synthesis activity. These results might be correlated with the downregulation of *MYH* and *OGG1* gene expression. These data are another evidence of the BER repair pathway impairment in Gorlin fibroblasts. In addition, ROS production and more specifically superoxide anion in Gorlin and normal fibroblasts demonstrate an increase after ionizing radiation. This correlates with our previous results that show a downregulation in *MYH* and *OGG1*. It has been shown that MYH-OGG1 deficient cells are sensitive to oxidants and ROS (42). As ROS accumulation could impair the activity of several important DNA repair proteins, we hypothesized that the increase in ROS production will lead to the increase of DNA damage. For example, OGG1 has critical redox-sensitive residues, and being at the reduced state, is important for its 8-oxoG DNA glycosylase activity. It has been shown that a polymorphic variant of OGG1 (serine to cysteine amino acid substitution at position 326) is associated with a higher risk of developing several types of cancer (43–45). The OGG1-Cys enzymatic activity decreases after a pro-oxidant treatment (46, 47). These findings support the important role of ROS in carcinogenesis since it not only induces oxidative DNA damage but also prevents its repair. Moreover, we have recently reported a general decrease in DNA repair under conditions leading to oxidative stress such as expression of amyloid β peptide (48), selenium depletion (30) or exposure to nanoparticles (49). We hypothesized the synergic effects of increased ROS production, accumulated oxidative DNA damage and impaired BER could participate to the hyper-radiosensitivity and cancer-prone phenotype of Gorlin patients where it is known that BER deficiency induces genetic instability and dramatic changes in gene expression, resembling changes found in many cancers (50). In the future, it will be important to decipher the oxidative status in NBCCS cell lines in order to explain the decrease of BER activity and the sensitivity of these cells to several oxidative stress agents such as H_2_O_2_ or KBRO_3_ and the modulation of antioxidant status (by measuring SOD, Catalase, GSH activities, lipid peroxidation, NRF2…).

## Data Availability Statement

All datasets generated for this study are included in the article/Supplementary Material.

## Author Contributions

AC wrote the first manuscript draft, performed the cell culture experiments, long term cytotoxicity assay, RT-qPCR experiments, mitochondrial superoxide assay, and performed the DNA excision-synthesis repair activity assay under the supervision and advice of SS. NF contributed in the writing and revision of the manuscript. TD and WR conceived the project, granted funding, supervised the project, and revised the manuscript. MM, PS, and SM provided cell strains, cell growth characterization (Supplementary Table S1 and responses to reviewers) and mutation sequencing (Supplementary Table S2 and responses to reviewers), and contributed to manuscript writing. SB-V, MS, SS, JL, and J-LR contributed with constructive comments on the manuscript. All authors read and approved the final version of the manuscript.

## Conflict of Interest

SS is employed by the company LxRepair. The remaining authors declare that the research was conducted in the absence of any commercial or financial relationships that could be construed as a potential conflict of interest.
