# Supplementary material for: Impairment of Base Excision Repair in Dermal Fibroblasts Isolated From Nevoid Basal Cell Carcinoma Patients
Source: Front Oncol. 2020 Aug 7;10:1551. doi: 10.3389/fonc.2020.01551 (PMC7427476; doi:10.3389/fonc.2020.01551)

**SUPPLEMENTARY DATA**

**Figure S1. Normal fibroblasts possessed the same sensitivity to different stresses.** Long term survival curves determined thanks to the clonogenic assay. Cells were plated at low density (20 cells/cm^2^) and the second day they were exposed to growing dose of **(A)** ionizing radiation, **(B)** UVA radiation, **(C)** UVB radiation or **(D)** bleomycin and cultured for 2 weeks. Cells were then fixed with ethanol, stained with crystal violet in order to count the number of colonies. Results are expressed as mean percentage of survival fraction ± SD of colonies of 3 independent experiments and triplicate measurements.

* significantly (p <0.05) different cells lines using Two-way Anova test.

**Figure S2. MTT assays demonstrate that this stress leads to less than 20% of mortality 24h after irradiation for both normal and Gorlin fibroblasts**

**Table S1:** Information on untransformed cells originating from Gorlin patients and healthy donors.

| **Cell strain** | **Sex**  **age, phototype, somatic origin** | **Origin**  **passage** | **Clinical data** |
| --- | --- | --- | --- |
| **GM02098**  fibroblast  untransformed | male, 31 years  caucasian  unknown | Coriell Institute  Camden, NJ, USA  frozen at p6 | Basal cell nevus syndrome BCNS  Clinically affected |
| **GM01552**  fibroblast  untransformed | male, 27  caucasian  skin | Coriell Institute  Camden, NJ, USA  p5 | Basal cell nevus syndrome BCNS  Clinically affected |
| **GM03300**  fibroblast  untransformed | female, 11  caucasian  unknown | Coriell Institute  Camden, NJ, USA  p2 | Basal cell nevus syndrome BCNS  Clinically affected |
|  |  |  |  |
| **GM00730**  fibroblast  untransformed | female, 45  caucasian  arm skin | Coriell Institute  Camden, NJ, USA  p6 | Healthy donor |
| **GM00116**  fibroblast  untransformed | female, 32  caucasian  mammary skin | MT Martin  CEA, Evry, France  p5 | Healthy donor |
| **GM00121**  Fibroblast  untransformed | female, 23  caucasian  mammary skin | MT Martin  CEA, Evry, France  p5 | Healthy donor |

**Genotyping analysis and characterization of mutations in Gorlin cell strains.**

539 *PTCH1* different mutations in 855 individuals have been thus far described for this rare genetic syndrome, which is notably characterized by a very large spectrum of heterozygous mutations in this gene (see LOVD data base, *PTCH1* data). Mutations in the *PTCH1* gene were characterized by NGS exome sequencing for the three cell lines studied and also by full genome sequencing for GM01552. Each Gorlin cell line harbors specific *PTCH1* heterozygous variants. GM01552 and 2098 have already been described in *Vulin et al., 2018*, we now add a new mutation for GM01552 and mutations for GM03300. Notably, a triplet insertion in 5’UTR was found in two cell lines, and this insertion was also found in another Gorlin patient (GM01575, Vulin et al. ). Using full genome sequencing; it would be interesting to investigate further this mutation in Gorlin syndrome, as it was found deleterious for transcript translation (Tietze, 2013).

**Table S2: Characterization of mutations in Gorlin cell strains.**

| **Cell line** | **Localization** | ***PTCH1* mutation** | **mRNA pattern** | **Predicted PTC1 protein impact** | **References** |
| --- | --- | --- | --- | --- | --- |
| GM01552 | 5‘UTR  98240652    5’UTR  98270647 | triplet insertion    triplet insertion  T>TGCC | triplet extension | unknown  expressed but functionally deficient | **unpublished***      Vulin, 2018 |
| GM02098 | Intron 17  98221881      5’UTR  98270647 | splice site mutation C>G      triplet insertion  T>TGCC | exon 17 skipping and stop codon in exon 18    triplet extension | truncated protein      expressed but functionally deficient | Chidambaram, 1996      Vulin, 2018  Tietze, 2013 |
| GM03300 | Exon 8   98241329  Exon 8  98241327 | mutation  frameshift | 1168 G>A  1170 delC stop codon in exon 9 | expressed but functionally deficient  truncated protein | **unpublished***  **unpublished*** |

Table legend: The three cell lines were sequenced by NGS to search for *PTCH1* gene mutations (exome or full genome), as described in *Vulin et al., IJROBP, 2018*. Specific heterozygous mutations were found, and also one triplet insertion in 5’UTR which was common to two cell lines. Localization is shown according to hg19. * new mutation, unpublished for any Gorlin patient. Cell lines from healthy donors were also sequenced, to check that *PTCH1* gene was wild type in these cells.

**Table S3. List of the primers used for the RT-qPCR experiments.** Reaction mix contains 10 ng of each cDNA, Takyon^TM^ qPCR MasterMix, and primers forward and reverse. qPCR was performed in an MX3005p Multiplex Quantitative PCR System (Stratagene, CA, USA) and the amplification’s integrity was checked at the end of each run.


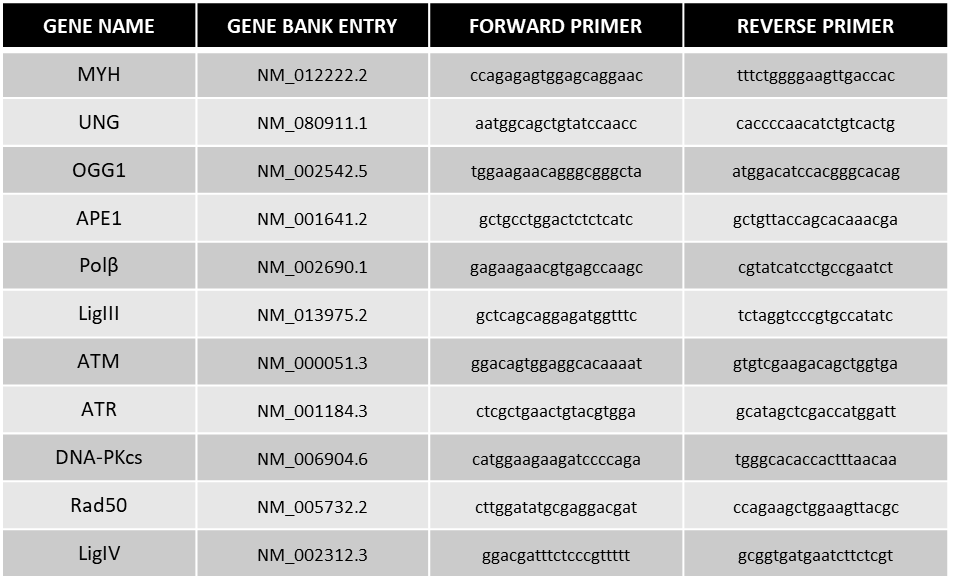


**Table S4. Determination of LD_50_ after exposure to ionizing radiation, UVA and UVB stress and bleomycin of control and Gorlin fibroblast using a clonogenic test.** LD_50_ determined the doses or concentration of a radiation or a compound that reduce in vitro cell survival by 50%. The results represent the average of three different experiments.


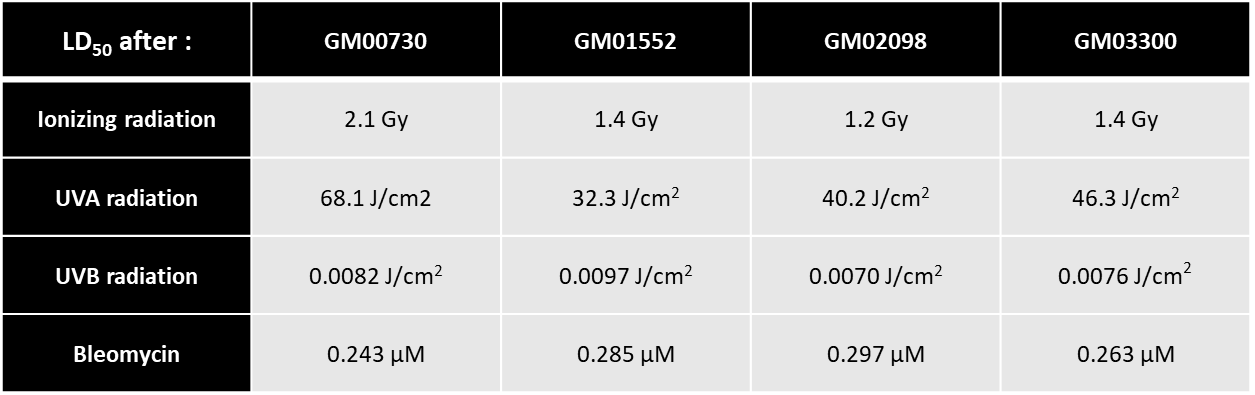

Supplement: Supplementary file 1 [file Data_Sheet_1.docx]
